# The in cellular and *in vivo* melanogenesis inhibitory activity of safflospermidines from *Helianthus annuus* L. bee pollen in B16F10 murine melanoma cells and zebrafish embryos

**DOI:** 10.1371/journal.pone.0325264

**Published:** 2025-06-24

**Authors:** Phanthiwa Khongkarat, Wannakarn Kitipaspallop, Songchan Puthong, Wittaya Pimtong, Preecha Phuwapraisirisan, Chanpen Chanchao

**Affiliations:** 1 Department of Biology, Faculty of Science, Chulalongkorn University, Bangkok, Thailand; 2 Institute of Biotechnology and Genetic Engineering, Chulalongkorn University, Bangkok, Thailand; 3 Nano Environmental and Health Safety Research Team, National Nanotechnology Center (NANOTEC), National Science and Technology Development Agency (NSTDA), Pathum Thani, Thailand; 4 Center of Excellence in Natural Products, Department of Chemistry, Faculty of Science, Chulalongkorn University, Bangkok, Thailand; Hirosaki University Graduate School of Medicine, JAPAN

## Abstract

Melanin, synthesized by tyrosinase (TYR), is a natural pigment essential for skin protection and pigmentation. However, excessive melanin production can cause dermatological disorders. Safflospermidines, comprised of safflospermidine A and B isomers isolated from sunflower (*Helianthus annuus* L.) bee pollen, were shown to exhibit a strong *in vitro* TYR inhibitory activity against mushroom TYR. However, their anti-melanogenesis activity in cellular and *in vivo* models remains unexplored. This study firstly evaluated the effects of these safflospermidines on melanogenesis in α-melanocyte stimulating hormone-stimulated B16F10 cells, using kojic acid as a positive control. Cytotoxicity was evaluated using the MTT assay, while TYR activity and melanin content were measured to assess melanogenesis inhibition. The expression of key melanogenesis-related genes was analyzed through quantitative real-time reverse transcription (RT-q)PCR to elucidate the molecular mechanisms involved. Secondly, the melanogenic activity and potential toxicity of the compounds were confirmed in zebrafish embryos, with phenylthiourea (PTU) as a reference. The results revealed that a mixture of these two safflospermidines exhibited no cytotoxicity across the treated concentration range (0–500 µg/mL). At 62.5 µg/mL, safflospermidines significantly reduced the intracellular melanin content by 21.78 ± 4.01% and the TYR activity by 25.71 ± 3.08% in B16F10 cells through downregulation of *TYR*, TYR-related protein 1 (*TRP-1*), and *TRP-2* gene expression. Additionally, the safflospermidines induced a noticeable reduction in dendritic cell structures, which likely contributed to the marked decrease in extracellular melanin levels. Kojic acid (250 μg/mL) significantly reduced the melanin content and TYR activity by suppressing all three melanogenesis-related genes. In zebrafish embryos, safflospermidines showed no toxicity or morphological abnormalities within a concentration range of 0–62.5 µg/mL, and at a concentration of 15.63 µg/mL the melanin production in zebrafish embryos was significantly reduced by 28.43 ± 9.17%. In comparison, 0.0015% (v/v) PTU decreased melanin production by 53.20 ± 3.75%. These findings suggest that safflospermidines are safe, effective melanin inhibitors with potential applications in pharmaceuticals and cosmetics for managing hyperpigmentation.

## 1. Introduction

Melanogenesis, the biological process responsible for melanin production, plays a central role in determining pigmentation in mammalian skin, hair, and eyes, while providing essential photoprotection against ultraviolet radiation (UVR) [1]. Melanin functions by absorbing UVR and neutralizing reactive oxygen species (ROS) generated during UV exposure, thereby preventing cellular damage [2]. Melanin synthesis is influenced by various factors, including genetic variations in the melanocortin-1 receptor (MC1R) gene, hormonal signals, cytokines, and environmental stimuli, such as UV exposure and oxidative stress. Among these, UVR is the most critical external factor affecting pigment formation [1,3]. These factors stimulate the secretion of α-melanocyte-stimulating hormone (α-MSH), which binds to MC1R on melanocytes and, via activation of intracellular signaling pathways, results in the upregulation of melanin production through the activation of key melanogenic enzymes [4]. Within melanocytes, melanogenesis occurs in specific organelles known as melanosomes, under the control of the microphthalmia-associated transcription factor (MITF), a master regulator of tyrosinase (TYR), tyrosinase-related protein 1 (TRP-1), and tyrosinase-related protein 2 (TRP-2). The TYR catalyzes the initial and rate-limiting steps in melanin synthesis by converting L-tyrosine to L-3,4-dihydroxyphenylalanine (L-DOPA) and subsequently oxidizing L-DOPA into dopaquinone, followed by the catalysis of TRP-1 and TRP-2 to produce eumelanin. Mature melanosomes are transported to keratinocytes to form a protective barrier against further UV-induced damage [5].

However, excessive melanin synthesis can result in hyperpigmentation disorders, such as freckles, melasma, post-inflammatory spots, and age spots, leading to cosmetic and therapeutic concerns. Therefore, TYR has become a primary target in the development of inhibitors to address these issues [6]. Current TYR inhibitors, such as hydroquinone, kojic acid, and arbutin, have notable side effects. Hydroquinone causes skin irritation, redness, and ochronosis with prolonged use, while kojic acid is unstable in light and heat, which reduces its effectiveness [7,8]. Although arbutin is safer, it can cause contact dermatitis and paradoxical hyperpigmentation at higher concentrations [9]. Thus, finding alternative TYR inhibitors with suitable safety and efficacy profiles is crucial in cosmetic applications.

Bee pollen, a natural product collected by bees, contains a diverse array of bioactive compounds, including proteins, vitamins, and phytochemicals [10]. It exhibits bioactive properties, including antioxidant, anti-inflammatory, antimicrobial, and antitumor effects [11–13]. The biological activities of bee pollen are influenced by its botanical origin, geographical source, and extraction methods [14,15]. Therefore, bee pollen has the potential to be a valuable source of TYR inhibitors, such as kaempferol and caffeine from Camellia pollen [16,17], and ergosterol peroxide from *Fagopyrum esculentum* bee pollen [15]. Furthermore, bee pollen-derived phenolamides have been reported as another class of TYR inhibitors. The structure of these compounds consists of hydroxycinnamic acids (such as *p*-coumaric, ferulic, and caffeic acids) linked to polyamines (such as spermidine, and spermine) through amide bonds [18]. For example, trisubstituted hydroxycinnamic acid spermidines obtained from the ethyl acetate fraction of Camellia bee pollen exhibited a 75% inhibition of TYR activity at a concentration of 0.5 mg/mL [19], while the phenolamides present in the ethyl acetate extracts of apricot bee pollen inhibited the mushroom TYR activity by over 50% at a concentration of 25 μg/mL [20].

Two phenolamides with mushroom TYR inhibitory activity were previously isolated from sunflower (*Helianthus annuus* L.) bee pollen collected in Thailand and identified as *N*^*1*^*,N*^*10*^*-(E)-N*^*5*^*-(Z)*-tri-*p*-coumaroyl spermidine (safflospermidine A) and *N*^*1*^*-(E)-N*^*5*^*,N*^*10*^*-(Z)*-tri-*p*-coumaroyl spermidine (safflospermidine B) [21]. Their TYR activity is related to the number and types of hydroxycinnamic acids linked to the polyamine backbone [22]. For instance, polyamines like spermidine or spermine bearing three to four hydroxycinnamic acids demonstrate the strongest inhibitory effects [23]. Safflospermidine A isolated from *Quercus mongolica* bee pollen in Korea displayed a notable TYR inhibitory activity [22]. Although these compounds exhibit a promising mushroom TYR inhibition, the anti-melanogenic potential of safflospermidines remains unexplored in cellular and *in vivo* models. Therefore, further investigation is required to ensure their potential as natural agents for hyperpigmentation treatment.

In this study, the melanogenesis inhibitory effects of safflospermidines in α-MSH-induced B16F10 cells, an *in vitro* model for melanogenesis research, was evaluated. The cytotoxicity of these compounds against B16F10 cells was determined using the 3-(4,5-dimethylthiazol-2-yl)-2,5-diphenyltetrazolium bromide (MTT) assay, and morphological changes were observed under light microscopy. Cellular TYR activity and melanin content were also measured and correlated with the expression levels of three melanogenesis-related genes using quantitative reverse-transcription polymerase chain reaction (RT-qPCR). In parallel, the toxicity and depigmentation effects of the safflospermidines were assessed in a zebrafish model to provide an *in vivo* perspective. The results of this study provide insights into the potential of safflospermidines as effective melanin-inhibiting agents and supports their development as natural candidates for managing hyperpigmentation in cosmetic and pharmaceutical applications.

## 2. Materials and methods

### 2.1. Compound preparation

**Fig 1:**
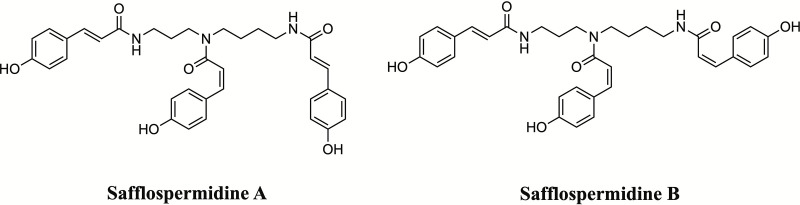
The chemical structure of safflospermidine A and B [21].

The sample for this experiment was a mixture of safflospermidine A and safflospermidine B, with their structures shown in Fig 1. Both compounds were isolated from sunflower (*Helianthus annuus* L.) bee pollen (SBP) collected in Lopburi province, Thailand (14°51’30.8“N, 101°01’07.5”E). The isolation and structure elucidation of these compounds were reported previously [21]. Briefly, the SBP was extracted with methanol (MeOH), and the resulting crude MeOH extract of SBP (CSBP) was sequentially partitioned with hexane and dichloromethane (DCM). The DCM-partitioned extract of CSBP (DCMSBP), which exhibited the highest *in vitro* inhibitory activity against mushroom TYR, was subjected to silica gel 60 column chromatography, yielding fraction # 5 (DCMSBP5) as the most active fraction. This fraction was further purified using high-performance liquid chromatography (HPLC), resulting in a mixture of two active isomers. Both isomers were characterized using nuclear magnetic resonance (NMR) spectroscopy and mass spectrometry (MS) and identified as safflospermidine A and safflospermidine B.

To prepare various concentrations of the safflospermidine A and B mixture, hereafter denoted as safflospermidines, the test sample was dissolved in dimethyl sulfoxide (DMSO) to a stock concentration of 100 mg/mL for cellular experiments and 200 mg/mL for *in vivo* experiments. The 100 mg/mL stock solution was then diluted with cell culture media (CM) to reach a non-cytotoxic concentration [DMSO < 1% (v/v)] [24] and the 200 mg/mL stock was diluted with egg water (60 µg/mL sea salt in RO water) to reach a non-toxic concentration for zebrafish embryos [DMSO < 0.5% (v/v)] [25].

### 2.2. Cell culture

The B16F10 murine melanoma cell line (ATCC No. CRL-6475) was obtained from the Institute of Biotechnology and Genetic Engineering, Chulalongkorn University. The cells were grown in CM [Dulbecco’s Modified Eagle’s Medium supplemented with 10% (v/v) fetal calf serum and 1% (w/v) penicillin/streptomycin] in a humidified environment with 5% CO_2_ at 37 °C. When the confluency reached 80%, the cells were detached from the culture flask using 0.05% (w/v) trypsin-EDTA and subcultured in fresh CM. The cells were then counted using a hemocytometer and seeded into culture plates for further experiments.

### 2.3. Cell viability assay

The viability of the B16F10 cells was determined using the MTT assay as previously described [26] with slight modifications. In brief, B16F10 cells (1 × 10^4^ cells/well) were cultured in a 96-well plate containing 200 μL of CM and allowed to adhere overnight. After that, the cells were treated with 2 µL/well of different concentrations of safflospermidines (3.91-500 μg/mL), kojic acid (positive control, 3.91-500 μg/mL), or DMSO solvent only (negative control); all treatments resulting in a 1% (v/v) DMSO concentration. The B16F10 cells were exposed to the treatment for 24, 48, and 72 h. At the end of the incubation period, 10 μL of 5 mg/mL MTT in normal saline solution was added to each well and then incubated at 37 °C for 4 h to allow formazan formation. The supernatant from each well was then removed, 150 μL of DMSO was added to dissolve the formazan crystals, and the absorbance at 540 nm (A_540_) was recorded using a microplate reader. The experiments were conducted in three independent replicates. The results are expressed as percentages of viability relative to the control, calculated using Eq. (1);


$$\begin{array}{c} \text{Relative\textbackslash{} cell\textbackslash{} viability}(\%)=\frac{{\text{A}}_{\text{sample}}}{{\text{A}}_{\text{control}}}\text{× 100} \end{array}$$ (1)

where A_sample_ and A_control_ represent the A_540_ values with and without the addition of test sample, respectively.

### 2.4. Cellular melanin content

Melanin content was determined as described previously [27] with slight modification. The B16F10 cells were seeded in 6-well culture plates (5 × 10⁴ cells in 2 mL of CM per well) and incubated overnight to allow the cells to adhere. After that, the CM was exchanged with fresh CM containing different concentrations of safflospermidines (62.5, 125, 250, and 500 μg/mL), kojic acid (250 μg/mL), or 1% (v/v) DMSO as a negative control, and in the presence of 100 nM α-MSH. The cells were then incubated for 72 h. For measuring the extracellular melanin content, the CM was collected and centrifuged at 1,500 × g for 10 min to remove cells and cell debris. Then, 200 μL of the supernatant was transferred into 96-well plates, and the absorbance was measured at a wavelength of 405 nm (A_405_). Subsequently, the intracellular melanin content was determined after removing the CM. The cells were first washed three times with phosphate buffered saline (PBS), then the cell pellets were solubilized by adding 500 μL of 1N sodium hydroxide (NaOH) containing 10% (v/v) DMSO and incubated at 80 °C for 1 h. The A_405_ was measured using a microplate reader. The experiments were carried out independently in triplicate. A standard curve of synthetic melanin was prepared, ranging from 0 to 300 μg/mL, to quantify the relative melanin content. The results were expressed as a percentage of the α-MSH-treated controls. In addition, the morphology of B16F10 cells in the treatment groups was observed under a light microscope at 200 × magnification.

### 2.5. Cellular TYR activity assay

Cellular TYR activity was determined as previously described [28] with minor modifications. B16F10 cells were seeded in 6-well culture plates at an initial density of 1 × 10^6^ cells/well in 2 mL of CM and incubated overnight to allow the cells to adhere. Subsequently, the CM was replaced with fresh CM containing various concentrations of safflospermidines (31.25, 62.5, and 125 μg/mL), kojic acid (250 μg/mL), or 1% (v/v) DMSO as a negative control, all in the presence of 100 nM α-MSH and cultured for 48 h. At the end of the respective treatments, the CM was discarded, and the cells were washed twice with ice-cold PBS, and then lysed in 400 µL of 0.01 M phosphate buffer (pH 7.4) containing 1% (v/v) Triton X-100 (lysis buffer). The mixture was freeze-thawed by incubating at −80 °C for 15 minutes, brought to room temperature for 10 minutes, and then centrifuged at 12,000 × g for 15 minutes at 4 °C. The supernatant was used to determine the protein concentration via a Bradford assay using bovine serum albumen as the standard, and protein concentrations were adjusted with lysis buffer. After that, in a 96-well plate, 100 μL of freshly prepared substrate solution (5 mM L-DOPA in 50 mM pH 7.1 sodium phosphate buffer) was added to the supernatant (100 μL), each containing 100 µg of protein. The mixtures were incubated at 37 °C for 1 h, and the absorbance was read at 475 nm (A_475_) using a microplate reader. The experiments were performed in triplicate. The percentage of TYR inhibitory activity was calculated using Eq. (2);


$$\begin{array}{c} \text{Cellular\textbackslash{} TYR\textbackslash{} inhibition}(\%)=\frac{{\text{A}}_{\text{control}}-{\text{A}}_{\text{sample}}}{{\text{A}}_{\text{control}}}\text{×}100 \end{array}$$ (2)

where A_sample_ and A_control_ indicate the A_475_ values with and without the addition of test sample, respectively.

### 2.6. Total RNA extraction

The B16F10 cells were seeded at a density of 1 × 10^6^ cells/well in 2 mL of CM in 6-well culture plates and incubated for 24 h. The CM was replaced with fresh CM containing safflospermidines (62.5 μg/mL), kojic acid (250 μg/mL), or 1% (v/v) DMSO as a negative control, all in the presence of 100 nM α-MSH for 48 h. After treatment, the cells were rinsed twice with ice-cold PBS and harvested using a cell scraper. The cell suspension was then centrifuged at 1,500 × g for 5 min, the supernatant was discarded, and total RNA was extracted from the cell pellet using the RNeasy mini kit (Catalog No. 74104; Qiagen, Valencia, CA, USA), according to the supplier’s protocol. The isolated RNA samples were quantified using a nanophotometer (NanoPhotometer NP80, Implen GmbH, Munich, Germany) and stored at −20 °C until RT-qPCR analysis.

### 2.7. Transcript analysis by RT-qPCR

The RT-qPCR was performed using the One-Step SYBR^®^ PrimeScript^TM^ RT-PCR Kit II (Perfect Real Time; Catalog No. R086A; Takara, Tokyo, Japan). Each reaction mixture, with a total volume of 10 µL, included 5 µL of 2x One-Step SYBR^®^ RT-PCR Buffer IV, 0.25 µL of PrimeScript^TM^ Enzyme Mix II, 0.5 µL each of the forward and reverse primers (10 µmol/L), and 2 µL of RNA template (20 ng). The final volume was adjusted with RNase-free water. The amplification conditions for the RT-qPCR assay consisted of a reverse transcription step at 50 °C for 2 min, followed by 95 °C for 10 min. The PCR amplification step was comprised of 40 cycles at 95 °C for 15 s and 50 °C for 1 min. The reaction was conducted on a QuantStudio™ 3 Real-Time PCR System (Thermo Fisher Scientific, Waltham, MA, USA). The primers for the target genes are shown in Table 1. *Glyceraldehyde 3-phosphate dehydrogenase* (*GAPDH*) was utilized as the internal standard. All reactions were conducted in triplicate. The 2^-ΔΔCT^ method was used to estimate the relative expression of target genes, normalizing it to the expression level of *GAPDH* and relative to the control group.

**Table 1:** Targeted genes and primers used for their amplification by RT-qPCR.

Gene	Forward primer (5’ to 3’)	Reverse primer (5’ to 3’)
*GAPDH*	GGGCATCCTGGGCTACTCTG	GAGGTCCACCACCCTGTTGC
*TYR*	GGGCCCAAATTGTACAGAGA	ATGGGTGTTGACCATTGTT
*TRP-1*	GTTCAATGGCCAGGTCAGGA	CAGACAAGAAGCAACCCCGA
*TRP-2*	GCTTGGAGCAGCAAGACAAG	ATTACACAGTGTGACCCGGC

### 2.8. Maintenance of zebrafish and embryo collection

Wild-type zebrafish (*Danio rerio*) were maintained at the National Nanotechnology Center. The adult zebrafish were housed in a standalone recirculating water system with specific parameters of a conductivity of 300–1,500 µS, pH of 6.8-7.5, and a light/dark cycle of 14/10 h. The room temperature was maintained at a constant 28 ± 1 ºC. Embryos were obtained through natural pair-wise mating. The animal procedures were performed in compliance with the NSTDA Institutional Animal Care and Use Committee regulations, following the animal study protocol No. 005–2562. Following the experiments, zebrafish embryos at 48 hpf were euthanized by submersion in an ice bath for at least 20 min to ensure cessation of all physiological activity.

### 2.9. Assessment of zebrafish embryo toxicity and melanin content

Healthy embryos were selected within 3 hours post-fertilization (hpf) and distributed into 24-well plates (30 embryos per well). The safflospermidines stock solution (200 mg/mL in DMSO) was diluted in egg water to achieve final concentrations of 0, 3.91, 7.81, 15.63, 31.25, and 62.5 µg/mL with 0.5% (v/v) DMSO. The exposure solutions were replaced every 24 h, and the embryos were continuously exposed for up to 48 h. Phenylthiourea (PTU) at 0.0015% (v/v) was used as a positive control to inhibit pigmentation. The survival rate at the endpoint was recorded using a stereomicroscope (SZX16, Olympus, Tokyo, Japan). Any experiments with control groups exhibiting a mortality rate exceeding 10% were excluded from the analysis. The experiments were conducted independently three times.

Following a 48-h exposure to the respective treatment, the melanin content in the zebrafish embryos was determined as previously published [29] with modifications. After documenting the phenotype of the embryos under a stereomicroscope, 30 embryos per test condition were rinsed with PBS to remove any extraneous material and then homogenized in a lysis buffer [Tris-PBS 98% (v/v), TritonX-100 1% (v/v), PMSF 1% (v/v)]. The homogenates were centrifuged at 10,000 × g for 5 min to isolate the melanin precipitate. The pellets were dissolved in 300 µL of 1 N NaOH for 1 h at 85 °C, centrifuged to obtain a clarified supernatant that was then transferred to a 96-well plate, and the absorbance at 490 nm (A_490_) was measured using a Spectramax iD5 microplate reader (Molecular Devices, USA). The A_490_ value correlates directly with the melanin content. Three independent replicates of these measurements were performed.

### 2.10. Statistical analysis

All data are presented as the mean ± one standard deviation (SD), derived from three independent repeats for each experiment. Significant differences between groups were analyzed using one-way analysis of variance (ANOVA) followed by Tukey’s or Dunnett’s T3 tests for pairwise multiple comparisons. Statistical significance was considered at the *p* < 0.05 (*), *p* < 0.01 (**), and *p* < 0.001 (***) levels. All statistical analyses were performed using IBM SPSS Statistics version 29.0.1.0 for Windows.

## 3. Results

### 3.1. Effect of safflospermidines on cell viability

**Fig 2:**
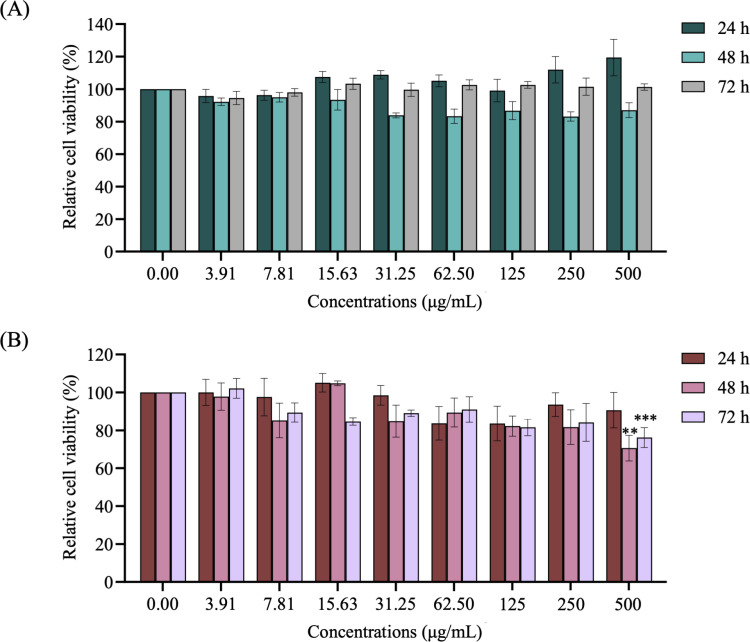
Relative cell viability of B16F10 cells following treatment with various concentrations of (A) safflospermidines and (B) kojic acid for 24, 48, and 72 h, as determined by the MTT assay. Data are presented as the mean ± SD from three independent experiments. Significant differences compared to the control group are shown as ** and *** for *p* < 0.01 and *p* < 0.001, respectively, (one-way ANOVA followed by Tukey’s post-hoc test for kojic acid-treated cells at all time points).

To evaluate the influence of safflospermidines and kojic acid on the viability of B16F10 cells, various concentrations were tested over 24, 48, and 72 h using the MTT assay. Safflospermidines at concentrations from 3.91 to 500 μg/mL exhibited no significant cytotoxic effects, maintaining a cell viability above 80% at all three time points compared to the control group (Fig 2). Similarly, kojic acid at concentrations between 3.91 and 250 μg/mL showed no cytotoxic effects at all three time points. However, higher concentrations of kojic acid resulted in significant cytotoxicity, with cell viability decreasing by 29.35 ± 6.72% at 48 h and 23.80 ± 5.21% at 72 h at a kojic acid concentration of 500 μg/mL. Consequently, for further studies on melanogenesis in B16F10 cells, the safflospermidines were used at concentrations ranging from 3.91 to 500 μg/mL while kojic acid at 250 μg/mL was used as the positive control.

### 3.2. Effect of safflospermidines on melanin content and cell morphology

**Fig 3:**
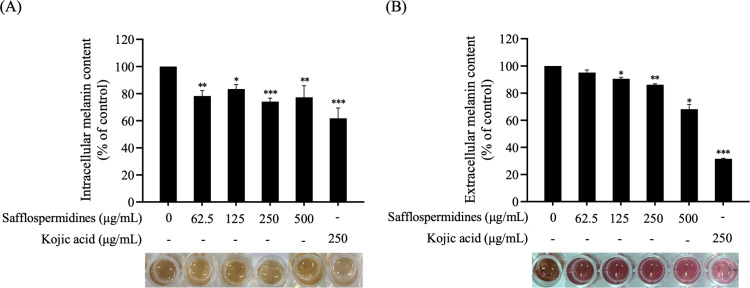
Effect of safflospermidines and kojic acid on B16F10 cells after 72 h treatment, showing the (A) intracellular and (B) extracellular melanin content. Melanin content is expressed as a percentage of the control group (set at 100%). Data are presented as the mean ± SD from three independent experiments. Significant differences compared to the control group are shown as *, **, and *** for *p* < 0.05, *p* < 0.01, and *p* < 0.001, respectively, (one-way ANOVA followed by Tukey’s post-hoc test for intracellular melanin and Dunnett’s T3 test for extracellular melanin). The bottom panel shows the color intensity of (A) dissolved melanin after cell lysis and (B) melanin secreted into the CM.

The effects of safflospermidines on melanin production were evaluated using both intracellular and extracellular melanin content assays. For this, B16F10 cells were treated with varying concentrations of safflospermidines (62.5−500 µg/mL) or kojic acid (250 µg/mL) for 72 h in the presence of 100 nM α-MSH. Melanin content was quantified based on a standard curve generated from synthetic melanin (y = 0.004x + 0.0398; R² = 1) and compared to the α-MSH-treated control cells. The intracellular melanin content revealed that safflospermidines significantly inhibited melanin production in a dose-independent manner, with reductions of 21.78 ± 4.01%, 16.61 ± 3.29%, 25.94 ± 2.67%, and 22.70 ± 8.55% at concentrations of 62.5, 125, 250, and 500 µg/mL, respectively. This inhibition was visually evidenced by the reduced intensity of the brown coloration, indicating suppressed melanin synthesis (Fig 3A). Similarly, extracellular melanin levels exhibited a somewhat comparable but dose-dependent reduction, with decreases of 4.85 ± 1.93%, 9.39 ± 1.02%, 13.76 ± 0.61%, and 31.82 ± 3.43% at concentrations of 62.5, 125, 250, and 500 µg/mL, respectively. The lighter color of the CM in the treated samples further supported a reduction in melanin release (Fig 3B). In contrast, 250 µg/mL kojic acid significantly decreased the intracellular and extracellular melanin content by 38.17 ± 7.60% and 68.47 ± 0.42%, respectively, as indicated by the diminished color intensity in both assays (Fig 3A, B).

**Fig 4:**
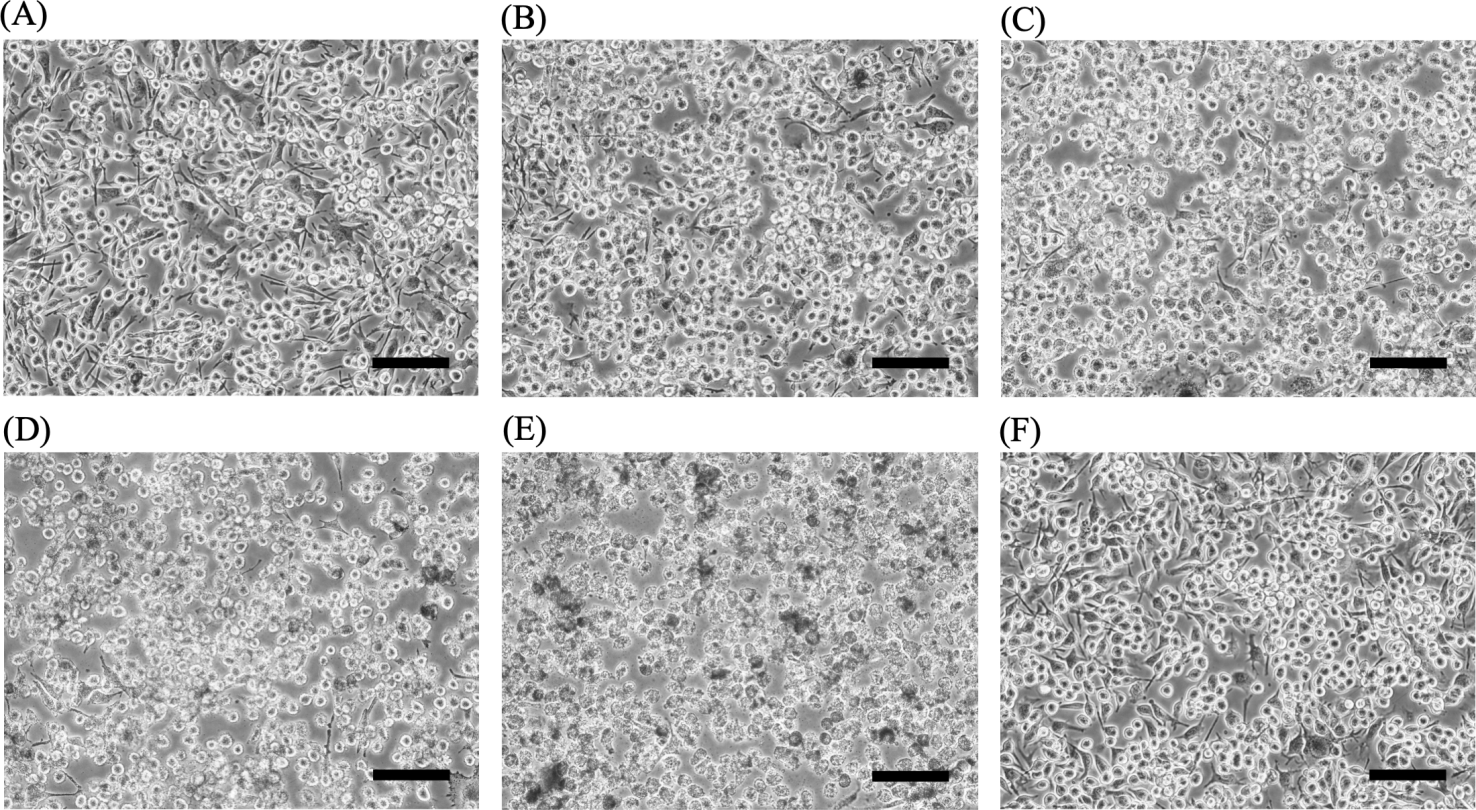
Morphology of B16F10 cells after 72 h of exposure to safflospermidines at (A) 0 μg/mL (control), (B) 62.5 μg/mL, (C) 125 μg/mL, (D) 250 μg/mL, and (E) 500 μg/mL, and (F) 250 μg/mL of kojic acid; as observed at 200X magnification under light microscopy. Each image is representative of three independent experiments. Scale bar = 50 μm.

The morphology of B16F10 cells was observed under light microscopy at 200X magnification. In the control group, the cells displayed a combination of spindle-shaped and epidermal-like structures, maintaining close contact with neighboring cells and adhering to the surface of the well plate (Fig 4A). Treatment with safflospermidines at 62.5 and 125 µg/mL induced noticeable morphological changes, including cell rounding and dendritic shortening compared to the control (Fig 4B, C). At higher concentrations (250 and 500 µg/mL), morphological assessment was limited due to the incomplete dissolution of the compound in CM, leading to precipitation. Nevertheless, a reduction in dendrite length was still observed (Fig 4D, E). In contrast, treatment with kojic acid at 250 µg/mL did not result in any significant morphological alterations (Fig 4F).

### 3.3. Effect of safflospermidines on cellular TYR inhibitory activity

**Fig 5:**
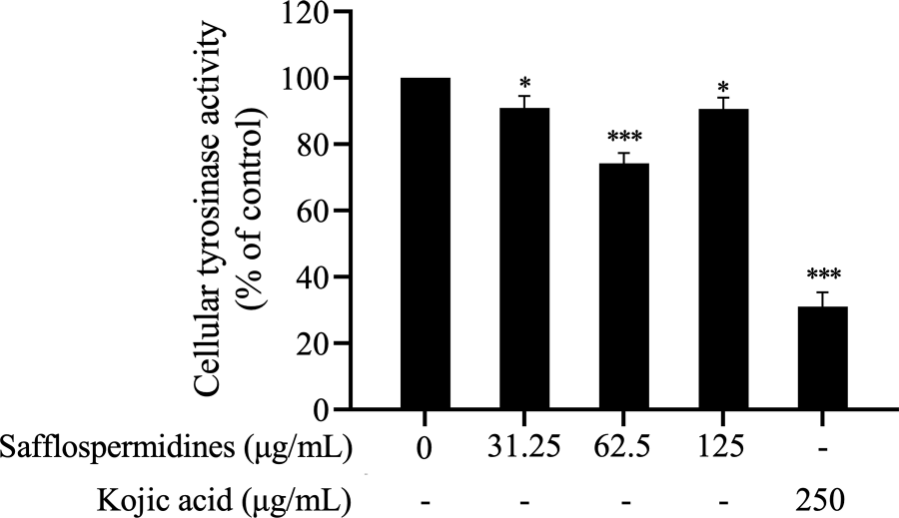
Effect of safflospermidines and kojic acid treatment for 48 h on the cellular TYR activity of B16F10 cells. Results are presented as the mean ± SD from three independent experiments. Significant differences from the control group are shown as * and *** for *p* < 0.05 and *p* < 0.001, respectively, (one-way ANOVA followed by Tukey’s post-hoc test).

The impact of safflospermidines on the cellular TYR activity in α-MSH-stimulated B16F10 cells was assessed. Cells were treated with varying concentrations of safflospermidines (31.25, 62.5, and 125 µg/mL) or kojic acid (250 µg/mL) for 48 h. As shown in Fig 5, safflospermidines significantly reduced the TYR activity compared to the untreated control (set at 100%), with inhibition rates of 9.03 ± 3.53%, 25.71 ± 3.08%, and 9.38 ± 3.42% at concentrations of 31.25, 62.5, and 125 μg/mL, respectively. Kojic acid at 250 µg/mL, used as a positive control, resulted in a 69.00 ± 4.30% inhibition of the TYR activity.

### 3.4. Effect of safflospermidines on the transcriptional expression of genes involved in melanogenesis

**Fig 6:**
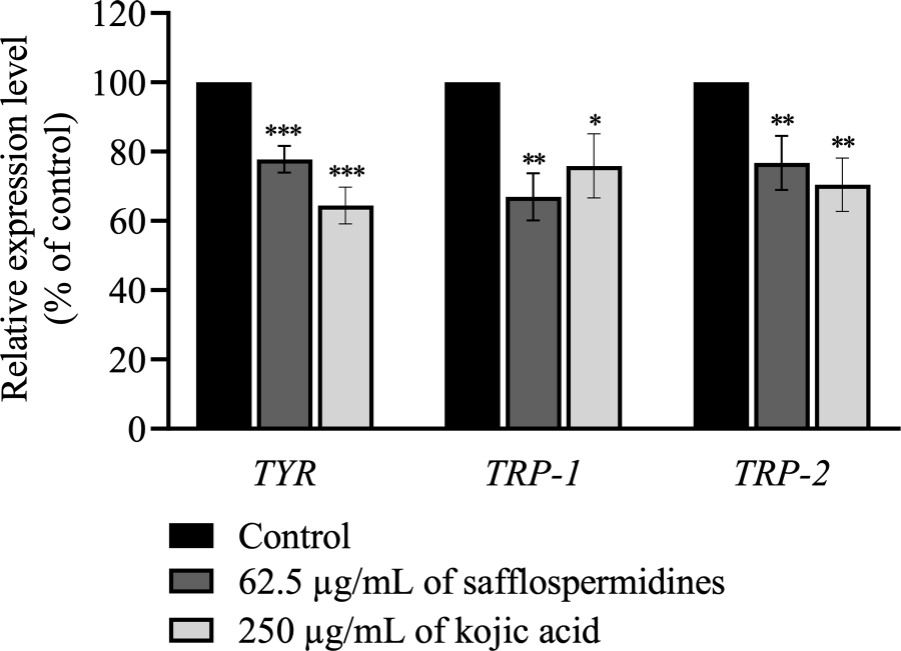
Effects of safflospermidines and kojic acid treatment for 48 h on the relative transcript levels of genes involved in melanogenesis in B16F10 cells. The mRNA expression level of *TYR*, *TRP-1*, and *TRP-2*, normalized to *GAPDH*, is shown relative to the untreated group. Results are presented as the mean ± SD of three independent experiments. Significant differences compared to the control group are shown as *, **, and *** for *p* < 0.05, *p* < 0.01, and *p* < 0.001, respectively, (one-way ANOVA followed by Tukey’s post-hoc test).

To investigate the effect of safflospermidines on the expression of melanogenesis-related genes, the mRNA levels of *TYR*, *TRP-1*, and *TRP-2* were measured in α-MSH-stimulated B16F10 cells using RT-qPCR. Since 62.5 µg/mL of safflospermidines significantly reduced the melanin content and TYR activity (Figs. 3 and 5), this concentration was selected for the gene expression analysis. The B16F10 cells were treated with either safflospermidines (62.5 µg/mL) or kojic acid (250 µg/mL) for 48 h. Following treatment, total RNA was extracted, and RT-qPCR was performed using gene-specific primers (Table 1), with *GAPDH* serving as the internal control. Safflospermidines (62.5 µg/mL) significantly reduced the expression level of *TYR*, *TRP-1*, and *TRP-2* by 22.21 ± 3.82%, 33.03 ± 6.80%, and 23.26 ± 7.79%, respectively, compared to control cells (Fig 6), while kojic acid (250 µg/mL), led to significant reductions in *TYR*, *TRP-1*, and *TRP-2* expression by 35.52 ± 5.31%, 24.11 ± 9.21%, and 29.55 ± 7.72%, respectively. These results suggest that safflospermidines and kojic acid both inhibit melanin synthesis in B16F10 cells by downregulating the transcription of *TYR*, *TRP-1*, and *TRP-2*.

### 3.5. Acute toxicity and antimelanogenic activity of safflospermidines in zebrafish embryos

**Fig 7:**
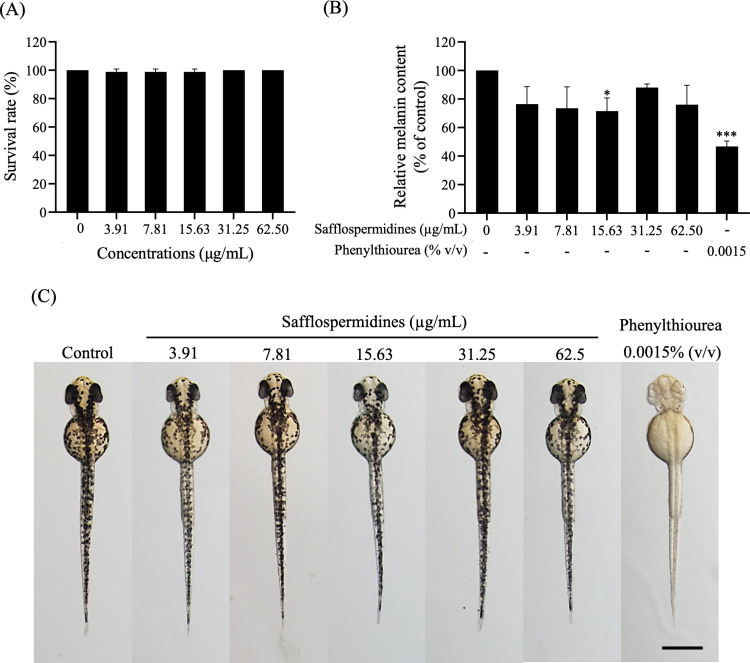
Effects of safflospermidines on zebrafish embryos: (A) Survival rate (%) and (B) relative melanin content (% of control) after exposure to safflospermidines at 3.91–62.50 µg/mL or 0.0015% (v/v) PTU. Significant differences from the control group are shown as * and *** for p < 0.05 and p < 0.001, respectively, (one-way ANOVA followed by Tukey’s post hoc test). (C) Dorsal view showing the morphology and melanin production after 48 h of treatment.

To assess the antimelanogenic effects of safflospermidines in zebrafish embryos, the acute toxicity was first evaluated by exposing embryos to safflospermidines over a concentration range from 0 to 62.5 µg/mL for 48 h. The survival rates across all treatment groups showed no significant differences compared to the control (Fig 7A). Additionally, all embryos showed no detectable morphological abnormalities (Fig 7C), indicating that safflospermidines did not induce acute toxicity at the tested concentrations. In terms of the melanin content, melanin precipitates were extracted from embryos across all experimental conditions. Zebrafish embryos treated with increasing concentrations of safflospermidines (3.91–62.50 µg/mL) exhibited a gradual decrease in their relative melanin content with a significant reduction of 28.43 ± 9.17% compared to the control group being observed at 15.63 µg/mL. Additionally, 0.0015% (v/v) PTU, as a positive control, significantly reduced melanin by 53.20 ± 3.75% compared to the control group (Fig 7B). This reduction in melanin was further supported by the visual assessment of the zebrafish embryos, where those treated with safflospermidines displayed a lighter pigmentation compared to the densely pigmented control group. In contrast, the positive control group treated with PTU exhibited complete inhibition of melanin synthesis, resulting in completely depigmented embryos (Fig 7C).

## 4. Discussion

Melanin, a dark pigment synthesized by melanocytes, plays a critical role in protecting the skin from UVR and reducing the effects of ROS [2]. However, prolonged UV exposure can cause excessive melanin production, leading to hyperpigmentation disorders. As a key enzyme in the melanogenesis pathway, TYR is a primary target for controlling abnormal melanin synthesis [6]. This has prompted the search for safe and effective anti-melanogenesis agents from natural sources that can inhibit TYR activity or regulate the genes involved in melanin production.

Safflospermidine A and B isolated from sunflower bee pollen were previously reported to exhibit a strong inhibitory activity against mushroom TYR *in vitro* [21]. However, the anti-melanogenesis effects of these compounds in B16F10 cells, their *in vivo* effects in zebrafish embryos, and their underlying molecular mechanisms have not yet been examined. In this study, a mixture of safflospermidine A and B was used without further purification due to their own geometric isomerism and ability to interconvert under light exposure, making it difficult to separate them [30]. The B16F10 cell line was used as a model due to its high melanin production, active TYR expression [31], and responsiveness to α-MSH or UVR, which closely resemble the melanogenic process in human melanocytes, as well as its ease of cultivation [32,33]. Kojic acid, a well-known TYR inhibitor, was used as a positive control due to its ability to inhibit both the monophenolase and diphenolase activities of TYR, reducing the intracellular melanin content in B16F10 cells without affecting cell viability at effective concentrations [34].

The cytotoxicity of safflospermidines on B16F10 cells was evaluated as a preliminary step. The results demonstrated that safflospermidines exhibited no cytotoxicity at concentrations up to 500 μg/mL, maintaining a relative cell viability above 80% [35], while kojic acid displayed cytotoxic effects at 500 μg/mL. These findings are consistent with previous studies, which reported that *N¹,N^5^,N^10^-(E)*-tri-*p*-coumaroyl spermidine was nontoxic to HepG2 cells at concentrations up to 500 μM and provided a protective effect on hepatocytes [36]. Additionally, rape pollen containing four phenolamines, with tri-*p*-coumaroyl spermidine as a constituent, has been shown to be nontoxic to HepG2 cells at concentrations up to 100 μg/mL [37]. Structurally, safflospermidines consist of a spermidine backbone conjugated to coumaric acid, both of which have been separately reported as nontoxic compounds to murine melanoma cells at concentrations up to 2,000 μM and 400 μg/mL, respectively [38,39] These findings demonstrated that safflospermidines are safer than kojic acid, even at higher concentrations.

In the melanogenesis study, 100 nM α-MSH was used to stimulate melanogenesis by activating MC1R, mimicking the cellular response to UVR. This concentration was deemed most appropriate as it minimizes factors that could interfere with cell proliferation and division [40]. The anti-melanogenesis activity of safflospermidines was evaluated in terms of the intracellular and extracellular melanin content. The safflospermidines suppressed the intracellular melanin levels significantly in dose-independent manner at concentrations of 62.5–500 µg/mL. This likely reflects that the compound has a limited solubility in CM [41] as well as the low lipophilicity of the amide bond present in their structure that limits its cellular permeability [42].

However, the extracellular melanin content was significantly inhibited in a dose-dependent manner by the safflospermidines at concentrations between 125 and 500 µg/mL, consistent with the reduction of dendritic cell development causing a lower melanin transport. These results are consistent with several compounds with a potent skin-whitening property that act through dendrite suppression. For example, Ginsenoside F1 reduced melanin secretion in α-MSH-induced B16F10 cells by 60% [43], pterostilbene inhibited melanocyte dendritic development and melanosome transport in B16F10 cells [44], while 3,4-dihydroxybenzalacetone shortened human epidermal melanocyte dendrites (HEMS) and inhibited both the expression of proteins involved in melanin synthesis and the export of melanosomes in HEMS and B16F10 cells [45].

Melanin is formed via TYR activity and the expression of TYR-related genes. In this study, the inhibitory effects of safflospermidines on cellular TYR were assessed at concentrations below 125 µg/mL to ensure their complete dissolution in CM. Safflospermidines at a concentration of 62.5 µg/mL effectively inhibited cellular TYR, which corresponded to the marked reduction in cellular melanin content, whilst significantly suppressing the expression of the melanogenesis-related genes *TYR*, *TRP-1*, and *TRP-2*.

However, for its practical use, an evaluation of the biological activity *in vivo* is a crucial step in determining the potential medicinal applications of safflospermidines. Zebrafish embryos are widely used for *in vivo* models of melanogenesis due to the similarity of the genes and melanogenesis pathways to those in humans. Additionally, zebrafish embryos are naturally transparent, allowing direct observation of the melanin pigments accumulated on the body surface, both visually and by microscopic analysis [46,47]. Thus, the *in vivo* inhibitory effects of safflospermidines on melanogenesis were confirmed using zebrafish embryos as a model in comparison with PTU, an effective TYR inhibitor in zebrafish, as the positive control [48]. The results showed that safflospermidines reduced melanin accumulation at all tested concentrations in a dose-independent manner, with no toxicity to zebrafish embryos in terms of their viability and phenotypic appearance, even at the higher concentrations of safflospermidines. A significant melanin reduction was particularly noticed at a safflospermidines concentration of 15.63 µg/mL, which was four-times lower than the effective concentration in B16F10 cells. This potentially illustrates that the zebrafish model is more sensitive to the anti-melanogenesis activity of safflospermidines than the B16F10 cell line.

The anti-melanogenesis efficacy of safflospermidines in those two models might be due to their *p*-coumaric acid moiety. Firstly, its structure is analogous to L-tyrosine and can function as a mixed type (for tyrosine) or competitive inhibitor (for L-DOPA) of human TYR [49]. In B16F10 cells, *p*-coumaric acid demonstrated a greater anti-melanogenesis efficacy than arbutin, a widely used positive control, by reducing both the cellular melanin content and the TYR activity [50]. Additionally, its effects are mediated through suppression of *TYR*, *TRP-1*, and *TRP-2* transcription via the MC1R/cAMP/PKA signaling pathway, leading to downregulation of MITF expression [51]. In zebrafish embryos, *p*-coumaric acid reduced both the melanin content and TYR activity without causing toxicity or morphological abnormalities [52]. Conversely, the spermidine part, a polyamine backbone, has been reported to increase melanin production in human melanocytes by stabilizing the TYR-related proteins (TRP-1 and TRP-2) through alterations in the protein degradation pathways, but does not affect melanogenesis-related gene expression [53]. Thus, the anti-melanogenic effects observed in this study are most likely attributed to the *p*-coumaric acid component. Although safflospermidines exhibit a lower efficacy than kojic acid and PTU in inhibiting melanogenesis, they offer a safer profile for skin cells.

In this regard, safflospermidines show some potential as natural anti-melanogenic agents due to their relative safety and effectiveness in inhibiting melanogenesis. Melanogenic-related genes, including *TYR*, *TRP-1*, and *TRP-2*, are master regulated by *MITF*. To further elucidate its mechanism, an investigation of *MITF* and genes associated with melanin transport is required at the cellular level. Furthermore, the TYR inhibitory activity and the expression of genes involved in melanin synthesis and transport need to be analyzed in zebrafish models. Moreover, improving the solubility and cell permeability of safflospermidines could enhance their bioactivity. Encapsulation presents a promising strategy to address these limitations, as it can improve compound stability and bioavailability at the targeted sites by enhancing skin permeation, penetration, and distribution [54]. For instance, ethosome-encapsulated protopanaxtriol saponins (PTS ethosomes) demonstrated a two-fold higher melanin reduction in a mouse model, even at lower concentrations [55]. This inspired us to implement encapsulation techniques for safflospermidines to enhance their anti-melanogenic properties which will be evaluated in future studies.

## 5. Conclusions

Safflospermidines isolated from sunflower bee pollen demonstrated non-cytotoxic effects in B16F10 cells while effectively inhibiting melanin production and secretion. This was achieved through direct inhibition of TYR activity, suppression of *TYR*, *TRP-1*, and *TRP-2* transcription, and reducing the dendritic structures of B16F10 cells. Moreover, safflospermidines reduced zebrafish pigmentation without causing toxicity or morphological abnormalities. To clearly understand the anti-melanogenic mechanisms of safflospermidines, genes involved in melanin synthesis and transport should be investigated. Additionally, improving the cell permeability of safflospermidines should be considered in further studies. These findings highlight the potential of safflospermidines to be developed as a natural skin-whitening agent for practical use in the pharmaceutical and cosmetic industries.
